# Tributyltin (TBT) induces oxidative stress and modifies lipid profile in the filamentous fungus *Cunninghamella elegans*

**DOI:** 10.1007/s11356-013-2375-5

**Published:** 2013-12-05

**Authors:** Przemysław Bernat, Ewa Gajewska, Rafał Szewczyk, Mirosława Słaba, Jerzy Długoński

**Affiliations:** 1 Department of Industrial Microbiology and Biotechnology, Faculty of Biology and Environmental Protection, University of Łódź, Banacha 12/16, 90-237 Łódź, Poland; 2 Department of Plant Physiology and Biochemistry, Faculty of Biology and Environmental Protection, University of Łódź, Banacha 12/16, 90-237 Łódź, Poland

**Keywords:** Organotins, Tributyltin, *Cunninghamella elegans*, Phospholipids, ROS, Lipidomics

## Abstract

To investigate the response of the tributyltin-degrading fungal strain *Cunninghamella elegans* to the organotin, a comparative lipidomics strategy was employed using an LC/MS-MS technique. A total of 49 lipid species were identified. Individual phospholipids were then quantified using a multiple reaction monitoring method. Tributyltin (TBT) caused a decline in the amounts of many molecular species of phosphatidylethanolamine or phosphatidylserine and an increase in the levels of phosphatidic acid, phosphatidylinositol and phosphatidylcholine. In the presence of TBT, it was observed that overall unsaturation was lower than in the control. Lipidome data were analyzed using principal component analysis, which confirmed the compositional changes in membrane lipids in response to TBT. Additionally, treatment of fungal biomass with butyltin led to a significant increase in lipid peroxidation. It is suggested that modification of the phospholipids profile and lipids peroxidation may reflect damage to mycelium caused by TBT.

## Introduction

Organotin compounds have been used as heat stabilizers in PVC, catalysts in chemical reactions, glass coatings, agricultural pesticides, biocides in marine antifoulant paints, wood treatments, and preservatives (Ishihara et al. 2012). Among them, tributyltin (TBT) is reported to be immuno-, neuro-, hepato-, nephro-, and gastrotoxic and it also causes testicular damage (Gupta et al. 2011). However, the mechanism(s) by which TBT induces toxicity have not been fully established. Liu et al. (2008) reported that the organotin induced oxidative damage to mice cells both in vivo and in vitro. Oxidative stress, which results from the overproduction of reactive oxygen species (ROS), causes cellular oxidative injury such as lipid peroxidation, protein oxidation, and DNA damage (Ishihara et al. 2012). Moreover, according to Chahomchuen et al. (2009), TBT induced apoptosis in *Saccharomyces cerevisiae*, which was associated with ROS production. Moreover, TBT altered lipids homeostasis by disturbing fatty acids composition (Bernat and Długoński 2007, 2012).

Although traditional lipid analysis, such as gas chromatography, is a powerful method for determination of fatty acyl heterogeneity, the information on the origin of the fatty acyl moiety is generally lost. Therefore, to define TBT toxicity towards the lipids of living cells, novel techniques using high-performance liquid chromatography (HPLC) and (tandem) mass spectrometry (MS/MS) should be used (Retra et al. 2008).

**Fig. 1:**
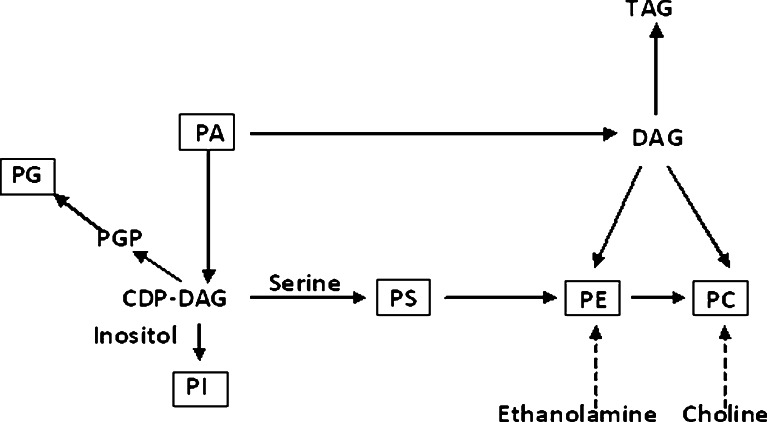
Representation of a metabolic network for phospholipids in *S. cerevisiae* (Xia and Yuan 2009). *CDP* cytidyldiphosphate, *DAG* diacylglycerol, *TAG* triacylglycerol, *PGP* phosphate-dylglycerophosphate

In the lipid fraction, phospholipids (PL) are considered to be the most sensitive to alterations in the physical environment (Dercová et al. 2004). PLs include primarily phosphatidylcholine (PC), phosphatidylethanolamine (PE), phosphatidylserine (PS), phosphatidylinositol (PI), lysophosphatidylcholine, lysophosphatidylinositol, phosphatidic acid (PA), and phosphatidylglycerol (PG). The synthesis of phospholipids in fungi occurs by complementary pathways common to those found in mammalian cells (Fig. 1) (Carman and Kersting 2004).

In our previous studies, we examined the TBT influence on fatty acids of the organotin metabolizing fungus *Cunninghamella elegans*. This strain presented high resistance towards TBT toxicity, surviving at the biocide concentrations of up to 20 mg l^−1^ (Bernat and Długoński 2002, 2006). The aim of this study was to expand our knowledge regarding the effects of TBT in *C. elegans* by PLs species analysis and determining the oxidative stress. This is also one of the first reports describing a simple method for the separation of molecular species of phospholipids from organic extracts of the filamentous fungus and their subsequent identification by the MS/MS technique in a negative ionization mode.

## Materials and methods

### Chemicals

TBT was purchased from Sigma-Aldrich (Poznan, Poland). 1,2-Dimyristoyl-sn-glycero-3-phospho-rac-(1-glycerol) (sodium salt) (14:0/14:0 PG); 1,2-dilauroyl-sn-glycero-3-phosphoethanolamine (12:0/12:0 PE); 1,2-dimyristoyl-sn-glycero-3-phosphocholine (14:0/14:0 PC); l-*R*-phosphatidylinositol (PI) sodium salt from bovine liver; 1,2-dimyristoyl-sn-glycero-3-phospho-l-serine sodium salt (14: 0/14:0 PS); and 1,2-dimyristoyl-sn-glycero-3-phosphate (sodium salt) (14:0/14:0 PA) were purchased from Avanti Polar Lipids (Alabaster, AL, USA). All of these compounds were added to a solution of mixed internal standards at the concentration of 0.1 mg ml^−1^ (IS solution). The other chemicals were from JT Baker (St. Louis, MO, USA), Fluka (Poznan, Poland), and POCh (Gliwice, Poland). All chemicals were high purity grade reagents. Stock solutions of TBT were prepared in 5 mg ml^−1^ ethanol.

### Microorganism

*C. elegans* (Lender) IM 1785/21Gp from the Department of Industrial Microbiology and Biotechnology fungal strain collection was the subject of our work. The features of this strain had been described in our earlier papers (Bernat and Długoński 2006, 2012).

### Growth conditions

Spores originating from 14-day-old cultures were used to inoculate 20 ml of Sabouraud dextrose broth liquid medium (Difco Laboratories, Detroit, MI, USA) (in 100 ml Erlenmeyer flasks). The cultivation was carried out at 28 °C on a rotary shaker (140 rpm) for 24 h. The precultures were transferred to fresh Sabouraud medium (in the ratio 1:4) and incubated for 24 h. Next, the mycelium was filtered and moved to the same volume of synthetic medium (Lobos et al. 1992). The medium consisted of (grams per liter): K_2_HPO_4_ (4.36), KH_2_PO_4_ (1.7), NH_4_Cl (2.1), MgSO_4_ × 7H_2_O (0.2), MnSO_4_ (0.05), FeSO_4_ × 7H_2_O (0.01), CaCl_2_ × 2H_2_O (0.03), glucose (40), and distilled water (up to 1,000 ml). Three milliliters of the homogenous preculture (mycelium in a synthetic medium) was introduced into 17 ml of synthetic medium with TBT (5 mg l^−1^) or without the organotin in the control cultures. The initial pH was 6.5. The cultures were incubated for 5 days. According to our previous study, the experiments were carried out in the exponential and stationary phases of growth (Bernat and Długoński 2012). Samples of fungal cultures at the exponential (24 or 48 h for control- and TBT-treated mycelium, respectively) and the stationary phase of growth (72 and 120 h for control and TBT, respectively) were used.

### Lipid extraction procedure

The resulting mycelium was filtered to dryness on Whatman #1 filter paper, weighed (wet weight), and then freeze-dried. Total lipids were extracted by the method of Folch et al. (1957) with modifications. Mycelia (~0.5 g) were crushed using a FastPrep-24 Instrument (MP Biomedicals, Santa Ana, CA, USA) homogenizer with 5 ml of a CHCl_3_–MeOH mixture (2:1, *v*/*v*) with 0.01 % butylated hydroxytoluene. The crushed cells were centrifuged at 1,000×*g* for 10 min at 4 °C. The supernatant was collected, and fresh 5 ml of the extraction mixture was added and vortexed for 1 min. After the mycelium had been centrifuged, both supernatants were combined with 2 ml of 0.8 % NaCl; the vials were vortexed for 1 min and centrifuged at 1,000×*g* for 5 min. The lower organic phase was collected, treated with anhydrous sodium sulfate, and evaporated under vacuum. The residue was then redissolved in 2 ml of methanol/CHCl_3_ (4:1, *v*/*v*) and stored at −20 °C pending analysis.

### Determination of PL molecular species by HPLC-MS/MS

Measurement of polar lipids was performed using an Agilent 1200 HPLC (Santa Clara CA, USA) system and a 3200 Q-Trap mass spectrometer (AB Sciex, Framingham, MA, USA) with an ESI source. For reversed-phase chromatographic analysis, 5 μl of the lipid extract were injected onto an Allure® PFP Propyl column (50 mm × 2.1 mm, 5 μm particle size; Restek, Bellefonte, PA, USA). The mobile phase consisted of 5 mM ammonium formate in water (A) and 5 mM ammonium formate in methanol (B). The solvent gradient was initiated at 80 % B, increased to 100 % B over 2 min, and maintained at 100 % B for two additional minutes before returning to the initial solvent composition over 2 min. The column temperature was maintained at 40 °C and the flow rate was 800 ml min^−1^. Prior to the next injection, a blank sample of methanol was run. The instrumental settings were as follows: spray voltage −4,500 V, curtain gas (CUR) 25, nebulizer gas (GS1) 50, turbo gas (GS2) 60, and ion source temperature of 600 °C. Data analysis was performed with the Analyst^™^ v1.5.1 software (AB Sciex, Framingham, MA, USA).

Tandem mass spectrometry for characterization/identification as well as quantification of lipid molecular species was performed using precursor ion scanning (Prec) and multiple reaction monitoring (MRM), respectively.

To perform the phospholipids species survey, an information-dependent acquisition method, Prec → EPI, was used. The spectra were obtained over a range from *m*/*z* 100 to 950. The EPI scan rate was 1,000 amu s^−1^. A scan of the precursor for *m*/*z* 153 was used to detect the subspecies of PA, PE, PI, PS, and scan of the precursor for *m*/*z* 168 was performed to find PC. The mass spectra of PA, PG, PI, and PE species showed ions corresponding to the deprotonated molecules [M-H]^−^.^.^In contrast, [M + HCOO]^−^ ions were observed for the charged PC species. Based on the product ion and precursor ion analysis of head groups and fatty acyls, a comprehensive list of MRM transitions was then generated to follow fatty acyl compositions of these lipids (parent R fatty acyl fragment transitions). The lipids belonging to each class were quantified upon comparison with the internal standard of the relevant class.

### Lipid peroxidation assay

The degree of lipid peroxidation was measured in terms of thiobarbituric acid-reacting substances (TBARS) content as described by Yagi (1976) with a modification (Słaba et al. 2013). The lyophilized biomass (30 mg) was mechanically homogenized (1:10 *w*/*v*) with a 50-mM sodium phosphate buffer (pH 7.0, temp. 4 °C) and centrifuged (24,000×*g*, 15 min). The supernatant was mixed with 29 mM thiobarbituric acid in 8.75 M acetate solution (1:1 *v*/*v*) and heated at 95 °C for 1 h. The cooled samples were supplied with *n*-butanol and intensively shaken. After centrifugation (10,000×*g*, 10 min), the fluorescence of the resulting organic layer was measured at 531 nm (excitation) and 553 nm (emission) using an F-2500 fluorescence spectrometer (Hitachi Ldt., Tokyo, Japan). The concentration of TBARS was estimated by referring to a standard 1,1,3,3-tetraetoxypropane. Finally, the level of lipid peroxidation was expressed as nanomoles of TBARS calculated per 1 g of lyophilized biomass.

### Data acquisition and multivariate statistical analysis

The experimental data represent the means of at least three independent experiments. Student's *t* test for unpaired samples after assuring homogeneity of variances was used to determine the significance of differences between the control and treatment mean values. Principal component analysis (PCA) was used to compare multivariate data from membrane lipid profiles. Data matrices with each column representing a specific PL molecule and each row representing a sample with a percentage value were analyzed. Because PE and PC were the major determined phospholipids and could cover other molecular species, each value for a certain species of PLs was divided by 100 % of the specific group of PLs it was in. PCA was performed using the MarkerView v1.2.1 software (AB Sciex, Framingham, MA, USA).

Double-bond index (DBI), which indicates the unsaturation level of lipids, was calculated by the equation: DBI = [sum of (*N* · × % lipid molecular species)]/100, where *N* is a number of double bonds in each lipid molecular species and % refers to % of a complex lipid class (Su et al. 2009).

## Results and discussion

### Phospholipids

The MS analyses provided information on molecular mass and PLs fragmentation. Using the LC-MS/MS procedure for a sample from the fungal strain, 49 species of PLs were identified, and the numbers of identified species for PA, PC, PE, PG, PI, and PS were 9, 8, 10, 3, 9, and 10, respectively (Table 1).

**Table 1:** Phospholipids identified in *C. elegans* and their MRM transitions

Lipids	MRM transitions		Lipids	MRM transitions	
*PA 16:0/18:3*	669.43	255	PS 18:3/18:3	778.45	277
*PA 16:0/18:2*	671.4	255	*PS 18:3/18:2*	780.37	277
*PA 16:0/18:1*	673.46	255	*PS 18:1/18:3*	782.48	277
*PA 18:3/18:3*	691.47	277	*PS 18:1/18:2*	784.51	279
*PA 18:3/18:2*	693.4	279	*PS 18:1/18:1*	786.57	281
*PA 18:3/18:1*	695.45	277	*PS 18:0/18:1*	788.49	283
*PA 18:1/18:2*	697.44	279	PS 18:0/18:0	790.57	283
*PA 18:1/18:1*	699.42	281	*PC 16:0/18:2*	802.5	279
*PA 18:1/18:0*	701.58	283	*PC 16:0/18:1*	804.5	255
*PE 16:0/18:2*	714.7	279	*PC 16:0/18:0*	806.5	255
*PE 16:0/18:1*	716.68	255	*PC 18:2/18:3*	824.5	279
*PE 18:2/18:3*	736.52	279	*PC 18:2/18:2*	826.5	279
*PE 18:1/18:3*	738.7	277	*PC 18:1/18:2*	828.5	279
*PE 18:2/18:2*	738.7	279	*PC 18:0/18:2*	830.5	279
*PE 18:1/18:2*	740.69	279	*PC 18:1/18:1*	830.53	281
*PE 18:1/18:1*	742.73	281	*PI 16:0/18:3*	831.65	255
*PE 18:1/18:0*	744.66	281	*PI 16:0/18:2*	833.6	255
PE 18:2/20:1	768.58	279	*PI 16:0/18:1*	835.73	255
PE 18:1/20:1	770.54	281	PI 18:3/18:3	853.5	277
PG 16:0/18:3	743.53	277	PI 18:2/18:3	855.66	277
PG 16:0/18:2	745.53	279	PI 18:2/18:2	857.55	279
PG 16:0/18:1	747.53	281	PI 18:1/18:2	859.55	279
*PS 16:0/18:3*	756.46	255	PI 18:1/18:1	861.56	281
*PS 16:0/18:2*	758.46	255	PI 18:0/18:1	863.59	281
*PS 16:0/18:1*	760.57	255			

The species which were used in quantitative analysis in this paper have been marked in italics

*PA* phosphatidic acid, *PC* phosphatidylcholine, *PE* phosphatidylethanolamine, *PG* phosphatidylglycerol, *PI* phosphatidylinositol, *PS* phosphatidylserine

Quantitative analysis of PLs for *C. elegans* revealed that PC constituted around 47–50 % of total cell PL in control samples and was a predominant PL in *C. elegans* (Table 2). Similarly, a previous study reported that PC was the most abundant phospholipid in *S. cerevisiae* (Xia and Yuan 2009). It was followed by PE which constituted around 35–39 % and PI and PS each accounting for about 4–8 %. The levels of the minor species, PA, were <3 %. Comparing samples from TBT medium with control medium, the strain exposed to TBT had significantly higher levels of PC (Table 2), whereas the control strain had higher levels of PE (Table 2). PC can be synthesized from PE, so they are closely related (Xia et al. 2011, Fig. 1). Because PE has a strong propensity to form non-bilayer hexagonal phases and PC is a bilayer-stabilizing lipid, the PC/PE ratio plays a key role in membrane integrity and cell function (Welti et al. 2002). Measurements of membrane competence showed that more ion leakage occurs in leaves, where the molar ratio PC/PE drops after freezing and which can lead to cell death (Welti et al. 2002). Due to the differences in the characteristics of PC and PE, the changes in PC/PE ratios in *C. elegans* from 1.42 to 1.93 for the control and TBT, respectively (for the stationary phase of growth), is indicative of a significant influence of the organotin on the membrane's composition.

**Table 2:** Comparison of phospholipid composition [percent] of *C. elegans* from exponential and stationary phases of growth exposed to TBT

Lipid species	Relative abundance [%]			
	Control		TBT	
	Exponential phase	Stationary phase	Exponential phase	Stationary phase
PA	1.33 ± 0.62	2.94 ± 0.02	9.4 ± 0.21*	5.02 ± 0.21*
PE	39.32 ± 3.57	35.03 ± 0.55	26.77 ± 5.32	29.25 ± 4.74
PS	6.22 ± 0.85	8.14 ± 0.98	1.47 ± 1.01*	4.64 ± 0.17*
PC	47.17 ± 4.7	49.96 ± 1.19	46.07 ± 5.87	56.6 ± 2.98
PI	6.01 ± 0.51	3.9 ± 0.1	16.25 ± 1.24*	4.48 ± 0.11*

Data are means ± SD

*P < 0.05 indicates values that differ significantly from the control

*PA* phosphatidic acid, *PC* phosphatidylcholine, *PE* phosphatidylethanolamine, *PI* phosphatidylinositol, *PS* phosphatidylserine

The other important lipid species include PI and PS. The syntheses of PI and PS are closely correlated because they both require CDP-DAG (Fig. 1). PS serves as a precursor to the synthesis of PE and PC (Xia et al. 2011). In mammalian and yeast cells, PS is normally distributed asymmetrically across the plasma membrane with the inner surface containing most of PS. The externalization of PS on the plasma membrane is an early indicator of apoptosis in mammalian cells (Delhaize et al. 1999). PI is essential for growth and metabolism of organisms. PI serves as a precursor for the synthesis of phosphoinositides, inositol polyphosphates, and complex sphingolipids and also plays an important role in the glycolipid anchoring of plasma membrane proteins (Gardocki et al. 2005). Therefore, regulation of the switch between PI and PS synthesis from CDP-DAG is critical. When comparing the PI and PS levels in the samples, we noticed that higher PI levels were negatively correlated with lower PS levels. It is possible that the higher PI/PS ratio could have resulted from declined synthesis of PSs or/and increased PIs synthesis in TBT presence.

PA, a lipid signal, normally constitutes a minor proportion of the cellular lipid pool, but in plants, in response to different biotic and abiotic stress factors such as bacterial and fungal pathogens, H_2_O_2_, chilling, salts, wounding, and heavy metals exposure, PA levels can increase significantly (Testerink and Munnik 2005; Darwish et al. 2009). In the present study, we found that the content of PA species increased almost 1.8-fold in mycelium exposed to TBT. Significant changes in PA species were accompanied by a lower level of PE in the presence of organotin. These results suggest possible conversion of PE species to PA via the phospholipase D pathway (Testerink and Munnik 2005).

**Fig. 2:**
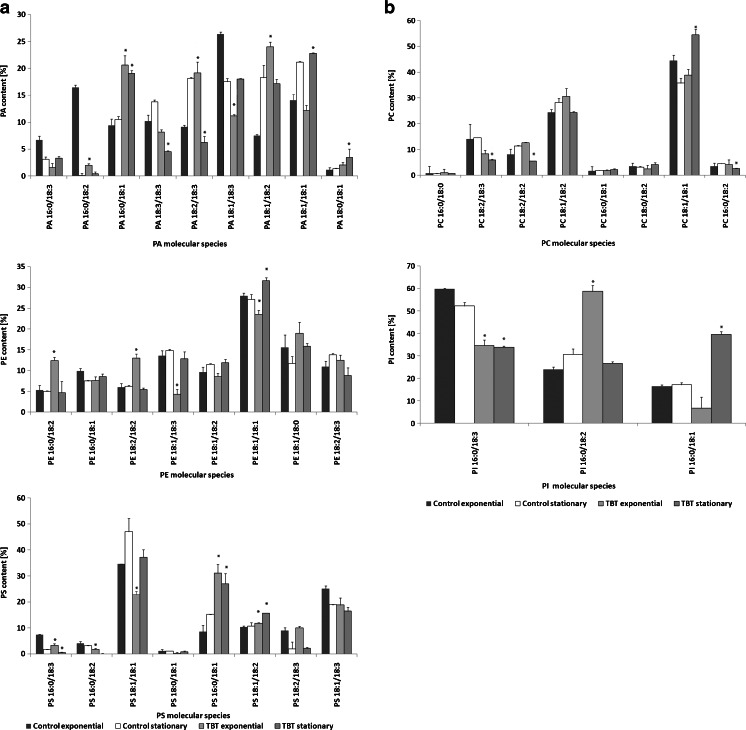
**a** Relative abundance for different PL molecules in the exponential and stationary phases of growth. *P < 0.05 indicates values that differ significantly from the control. *PA* phosphatidic acid, *PE* phosphatidylethanolamine, *PS* phosphatidylserine. The results are mean values of three experiments with standard deviations. **b** Relative abundance for different PC and PI molecules in the exponential and stationary phases of growth. *P < 0.05 indicates values that differ significantly from the control. *PC* phosphatidylcholine, *PI* phosphatidylinositol. *Error bars* represent standard deviations (*n* = 3)

Analysis of the distribution of the hydrocarbon chains showed that palmitic acid (C16:0) and stearic acid (C18:0) were the most predominant saturated fatty acids, whereas oleic acid (C18:1), linoleic acid (C18:2), and γ-linolenic acid (C18:3) were the major unsaturated species (Fig. 2a, b). This was in accordance with our previous determination of *C. elegans* fatty acids cultivated in Sabouraud or synthetic medium using the utilized GC/MS technique here (Bernat and Długoński 2007, 2012; Bernat et al. 2009). The mycelium exposed to TBT in the tested phases of growth exhibited a significant decline in the content of most phospholipid species containing C18:3: PA 16:0/18:3, PA 18:1/18:3, PA 18:3/18:3, PE 18:1/18:3, PS 16:0/18:3, PC 18:2/18:3, and PI 16:0/18:3. These results are consistent with our previous findings suggesting that the exposure of *C. elegans* to TBT leads to a decrease in the amounts of C18:3 fatty acid in glyco-, phospho-, and neutral lipids (the results based on the fatty acid methyl esters derivative analyses using GC/MS) (Bernat and Długoński 2012). In the presence of TBT changes were observed in the C18:2 fatty acid content. The amounts of phospholipids containing two C18:2 acyl species, such as PE 18:2/18:2 and PC 18:2/18:2, tended to increase in the exponential phase of growth. On the contrary, in the stationary phase of growth, the levels of both species declined (Fig. 2a, b). In our previous papers, we observed changes in the C18:0/18:1 ratio of TBT-treated fungal cells (Bernat et al. 2009; Bernat and Długoński 2012). Therefore, we summarized the C18:0 and C18:1 amounts in all determined phospholipids species and compared the outcomes with the results of traditional analysis. Using the ESI-MS/MS technique, the increased C18:0/18:1 ratio in TBT presence was also observed.

In fungal cells, unsaturated fatty acids are produced through desaturation of their saturated precursors. Subsequent desaturation of stearic acid by the action of Δ9, Δ12, and Δ6 desaturases leads to the production of C18:3 (Certik and Shimizu 1999). The decrease in the ratio of C18:3 to C18:2 or C18:1 might suggest the inhibition of desaturase activities in the presence of TBT.

To identify the possible roles of phospholipid fatty acids in TBT tolerance mechanisms in *C. elegans*, we used a DBI index, which indicates the level of lipids unsaturation (Table 3). In the stationary phase of growth, the DBI of PLs species in TBT-exposed cells was significantly lower than in control samples. These changes in DBI within fungal cultures were mainly caused by a decreased content of most phospholipid species containing C18:3 and increased amounts of less unsaturated species PA 16:0/18:1, PA 18:0/18:1, PE 18:0/18:1, PS 16:0/18:1, and PC 18:1/18:1 in the samples treated with TBT (Fig. 2a, b). Lower DBI indicates higher saturation of fatty acids. This is consistent with our previous studies of the *C. elegans* fatty acids profile (Bernat and Długoński 2007, 2012). We showed that the fatty acid unsaturation index was decreased, and the conversion of stearic acid (18:0) into oleic acid (18:1) was significantly inhibited in the presence of TBT. Moreover, the resulting membrane properties alteration led to a reduced permeability for K^+^ (Bernat et al. 2009). It seems that decreased PL unsaturation in TBT-exposed *C. elegans* leads to a decrease in its membrane fluidity (Hosono 1992; Kraft and Moore 2003; Turk et al. 2004). On the other hand, the increased PC/PE ratio promotes bilayer formation.

**Table 3:** DBI of lipid species of *C. elegans* exposed to TBT

Lipid species	DBI			
	Control		TBT	
	Exponential phase	Stationary phase	Exponential phase	Stationary phase
PA	3.23 ± 0.13	3.48 ± 0.11	3.14 ± 0.11	2.75 ± 0.12*
PE	2.56 ± 0.05	2.77 ± 0.08	2.53 ± 0.14	2.5 ± 0.06*
PS	2.85 ± 0.16	2.4 ± 0.01	2.51 ± 0.11	2.27 ± 0.04*
PC	2.79 ± 0.1	2.91 ± 0.02	2.77 ± 0.06	2.49 ± 0.12*
PI	2.43 ± 0.01	2.34 ± 0.01	2.27 ± 0.07*	1.94 ± 0.02*

Data are means ± SD

*P < 0.05 indicates values that differ significantly from the control

*PA* phosphatidic acid, *PC* phosphatidylcholine, *PE* phosphatidylethanolamine, *PI* phosphatidylinositol, *PS* phosphatidylserine

### Multivariate statistical analysis using PCA

**Fig. 3:**
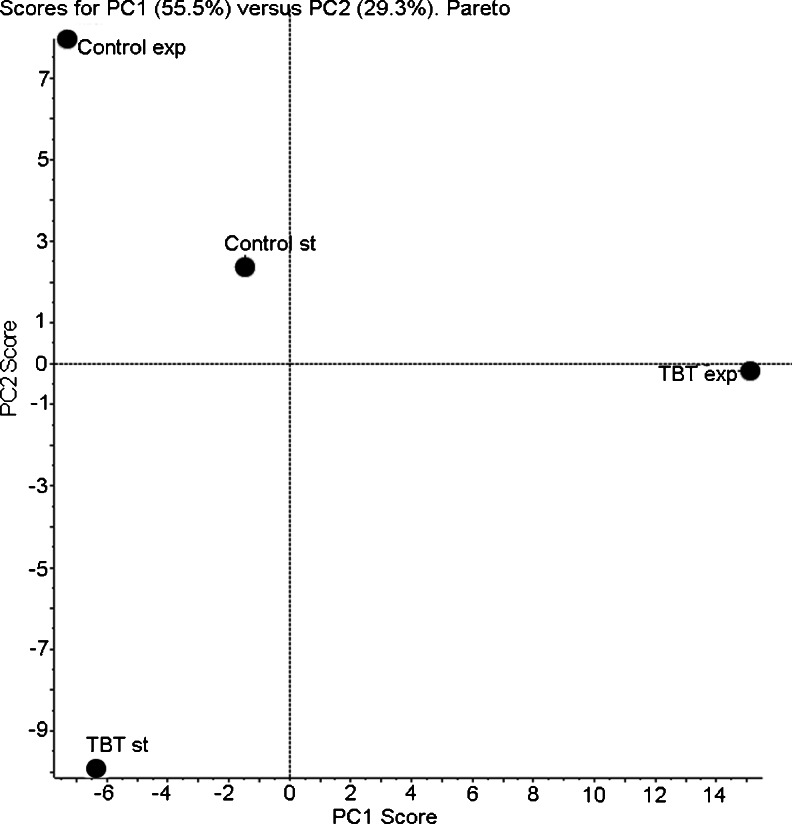
PCA of 36 lipid molecular species in *C. elegans* mycelium exposed to TBT. Principal components PC1 and PC2 accounted for 55.5 and 29.3 %, respectively, of the variance of the dataset for a total of 84.8 %

There were 36 variables (36 species of lipids) to describe the abundance of each lipid in each culture extract. PCA was used to transform these variables into 36 principal components, the first two of which accounted for most of the variance in the data. The score plot from the PLs of *C. elegans* showed differences in samples (Fig. 3). The PCA analysis revealed a clear separation among the growth states, especially in TBT-treated samples.

### Lipids of *C. elegans* peroxidation

**Fig. 4:**
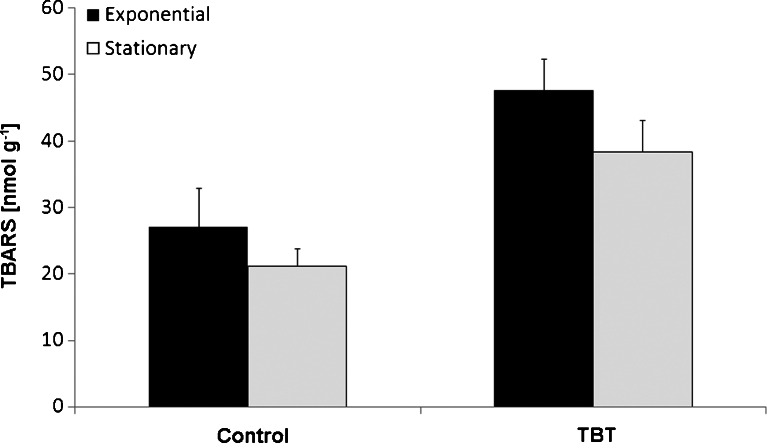
Levels of TBARS determined in *C. elegans* biomass withdrawn from the exponential and stationary phases of culture growth conducted in synthetic medium supplemented with TBT at the initial concentration of 0.0 or 5 mg l^−1^. *Error bars* represent standard deviations (*n* = 3)

Control biomass exhibited a measurable content of lipid peroxidation products, decreasing slightly in the course of the exponential/stationary phase of growth from 27.1 to 21.2 nmol TBARS g^−1^ lyophilized cells, respectively (Fig. 4). The levels of TBARS in biomass treated with TBT were statistically significantly higher (*P* < 0.05) than in control mycelium. In the exponential and stationary phase, the exposure to the organotin caused an increase in the mycelia TBARS levels by 71 and 81 % over the control, respectively.

The increased TBARS level found in TBT-treated *C. elegans* implies the induction of oxidative stress, which results from the imbalance between the generation of ROS and their removal by the antioxidative system of the cell. Also, according to Ishihara et al. (2012), the levels of TBARS in the rat organotypic hippocampal slices treated with TBT were approximately three times higher than those in untreated slices. Exposure to TBT with graded sublethal doses (0–200 nM) resulted in the release of ROS in the nematode *Caenorhabditis elegans* (Wang et al. 2012). Several studies have shown that the generation of ROS, including the species derived from H_2_O_2_ such as OH·, is a major mechanism of TBT toxicity (Gupta et al. 2011; Zhang et al. 2012). Although during *C. elegans* growth, TBT was converted into DBT and MBT (Bernat and Długoński 2006), a high level of TBARS in the stationary phase of growth was still observed. Therefore, we assumed that DBT could be responsible for the lipid peroxidation occurring in that phase. DBT induced an acute pancreatitis flare in rats through the generation of reactive oxygen species, and lipid peroxidation products increased within the pancreatic tissue exposed to the organotin (Lu et al. 2007).

In our study, TBARS and PA levels were significantly (*P* < 0.05) elevated in TBT-treated fungal cells. It is hypothesized that there were some connections between PA and ROS signaling in *C. elegans* exposed to TBT. However, according to our knowledge, there is no information about this phenomenon in fungal cells, and to date, only interactions between lipid species and ROS in plants were described by others. For example, the increase in PA in tomato has been linked to the initiation of ROS generation, a central event in plant resistance (Han and Yuan 2009). Additionally, exogenously applied PA has been shown to induce the formation of ROS in *Arabidopsis* sp. (Testerink and Munnik 2005). According to Gajewska et al. (2012), an exposure of wheat seedlings to Ni leading to a significant decrease in the level of linolenic acid (C18:3) could be partly related to lipid peroxidation. Considering the results obtained in this study, it is similarly possible that the decline in the polyunsaturated C18:3 content could be due to its oxidation.

## Conclusion

The data presented in this study demonstrated that TBT significantly influenced the phospholipid profile of *C. elegans* by modifying fatty acids content and classes of lipids. We also observed an increased level of lipids peroxidation. Thus, it cannot be excluded that ROS generation in fungal cells is correlated with an elevated concentrations of PA.

Although the genus *Cunninghamella* contains species of importance in medical mycology and in biotechnological processes (Asha and Vidyavathi 2009), to the best of our knowledge, this is the first report on the *Cunninghamella* phospholipids profile determined by use of the LC-MS/MS technique.
